# Krüppel-Like Factor 4 Inhibits the Transforming Growth Factor-*β*1-Promoted Epithelial-to-Mesenchymal Transition via Downregulating Plasminogen Activator Inhibitor-1 in Lung Epithelial Cells

**DOI:** 10.1155/2015/473742

**Published:** 2015-12-29

**Authors:** Fang Sun, Ke Hu

**Affiliations:** Division of Respiratory Disease, Renmin Hospital of Wuhan University, Wuhan 430060, China

## Abstract

Transforming growth factor-*β* (TGF-*β*) signaling and TGF-*β*-promoted epithelial-to-mesenchymal transition (EMT) have been postulated to be the common pathway causing pulmonary fibrosis. However, the up- or downstreaming markers of TGF-*β*-induced EMT still need to be further recognized. In the present study, we investigated the regulation on Krüppel-like factor 4 (KLF-4) and plasminogen activator inhibitor-1 (PAI-1) by TGF-*β* in the murine lung epithelial LA-4 cells and then examined the regulation of both markers in the TGF-*β*-induced EMT by the PAI-1 knockdown or the KLF-4 overexpression. Our study indicated that TGF-*β* induced EMT in mouse LA-4 lung epithelial cells via reducing E-cadherin, while promoting Collagen I and *α*-SMA. And PAI-1 was upregulated, whereas KLF-4 was downregulated in the TGF-*β*-induced EMT model in LA-4 cells. Moreover, the siRNA-mediated PAI-1 knockdown inhibited the TGF-*β*-induced EMT, whereas the adenovirus-medicated KLF-4 overexpression markedly reduced the PAI-1 expression and inhibited the TGF-*β*-induced EMT in LA-4 cells. In conclusion, our study confirmed the downregulation of KLF-4 in the TGF-*β*-induced EMT in LA-4 cells. And the KLF-4 overexpression significantly reduced the TGF-*β*-induced PAI-1 and thus inhibited the TGF-*β*-induced EMT in mouse lung epithelial LA-4 cells. It implies that KLF-4 might be a promising target for effective control of the pulmonary fibrosis.

## 1. Introduction

Pulmonary fibrosis, particularly idiopathic pulmonary fibrosis (IPF), is still an aggressive, fatal disease in adult humans because of the clinical and therapeutic dilemma for it. The pathogenesis of it is hypothesized as unknown environmental and/or occupational factors such as smoking, infection, or even tractional injury to the peripheral lung [1, 2]. Transforming growth factor-*β* (TGF-*β*) signaling has been widely postulated to be excessively activated as the final common pathway causing fibroproliferative disease and fibrosis including the pulmonary fibrosis [3, 4], via Smad3 signaling [5]. However, the function of TGF-*β* signaling is not only compartment specific but also temporally specific within the epithelial, mesenchymal, and immune components of the lung and the amelioration of IPF remains challenging by the blockage of TGF-*β* signaling. Therefore, other markers up- or downstreaming the TGF-*β* signaling in IPF need to be further recognized.

Krüppel-like factors (KLFs) are a family of zinc-finger transcription factors which are widely expressed in multiple human organs or tissues. KLFs pose regulatory roles in a variety of cellular processes such as proliferation, inflammation, differentiation, and migration [6]. The tumor suppressive KLF-4 has been identified to be decreased or lost in lung cancer [7] and other types of cancers [8–11]. And the inhibited KLF-4 results in epithelial-to-mesenchymal transition (EMT) by regulating the expression of EMT-related markers, such as E-cadherin, vimentin, *β*-catenin, vascular endothelial growth factor- (VEGF-) A, endothelin-1, and JNK1, or through crosstalk with the TGF-*β*, Notch, and Wnt signaling pathways [12, 13]. And a recent study has demonstrated that KLF-4 is downregulated during EMT in renal fibrosis and functions as a suppressor of renal fibrogenesis [14]. And the anti-EMT effect of KLF-4 has also been recognized in hepatocellular carcinoma cells [15].

Plasminogen activator inhibitor type-1 (PAI-1) is highly induced in epithelial cells undergoing an EMT-like conversion upon expression of the E-cadherin transcriptional repressors [16]. PAI-1 often localizes in tumor cells and myofibroblasts [17], implying involvement as a positive modulator of cellular invasive potential [18, 19]. PAI-1 is a target gene of TGF-*β* and Smads (Smad3 and Smad4) [20, 21]. And PAI-1 is regulated by TGF-*β* in different kinds of cells, such as macrophages [22], renal tubular cells [23], trophoblast cells [24], and hepatocytes [25]. PAI-1 has been recognized to be increased in pulmonary fibrosis [26], and animal studies have shown that experimental manipulations of PAI-1 levels directly influence the extent of scarring that follows lung injury [27–29]. However, it is not clear whether there is an interaction between KLF-4 and PAI-1 in the pulmonary fibrosis.

In the present study, we investigated the regulation on KLF-4 and PAI-1 by TGF-*β* in the murine lung epithelial LA-4 cells, then examined the expression of EMT-associated markers, such as Collagen I, *α*-SMA, and E-cadherin in the TGF-*β*-treated LA-4 cells, and determined the influence on the TGF-*β*-induced EMT by the PAI-1 knockdown or the KLF-4 overexpression. Our study indicated the inhibitory role of KLF-4 in the TGF-*β*1-promoted EMT via downregulating PAI-1 in the murine lung epithelial cells.

## 2. Materials and Methods

### 2.1. Cell Culture, Treatment, and PAI-1 Knockdown

Mouse lung epithelial LA-4 cell line was purchased from American Type Culture Collection (ATCC) (Rockville, MD, USA) and was grown in the F-12K Medium (Kaighn's Modification of Ham's F-12 Medium; GIBCO, Rockville, MD, USA), which was supplemented with 10% Fetal Bovine Serum (FBS; Invitrogen, Carlsbad, CA, USA) and with 100 U/mL penicillin and 100 mg/mL streptomycin (CSPC Pharmaceutical Group Limited, Shijiazhuang, China) at 37°C in a humid incubator. To model the TGF-*β*-induced EMT in LA-4 cells, LA-4 cells with approximately 90% confluence were incubated with the F-12K Medium, adding 2% FBS, and the TGF-*β* with a final concentration of 0, 1, 2, 5, or 10 ng/mL for 1, 3, 6, 12, 24, or 48 hours at 37°C. And then the cells were collected for Western blot assay or for real-time quantitative PCR (RT-qPCR) analysis. To knock down the PAI-1 expression, the PAI-1-specific siRNA (siRNA-PAI-1) or control scramble siRNA (siRNA-Con) was transfected with Lipofectamine RNAiMax (Invitrogen, Carlsbad, CA, USA) into LA-4 cells with a concentration of 20 or 40 nM to abrogate the PAI-1 expression.

### 2.2. Adenovirus-Mediated Overexpression of KLF-4 in LA-4 Cells

The open reading frame (ORF) of mouse KLF-4 was amplified via PCR with primers that deleted the stop codon and was inserted into the pShuttle-CMV vector to construct the recombinant pShuttle-CMV-KLF-4 plasmid. The chloramphenicol acetyl transferase (CAT) gene was utilized as a control. The adenovirus Ad-KLF-4 and the control Ad-Con virus were rescued by cotransfecting both the pShuttle-CMV-KLF-4 and the pAdEasy-1 (harboring the genomic sequence of adenovirus) into BJ5183 bacterial cells. To investigate the promotion to KLF-4 in LA-4 cells, approximately 90%-confluent LA-4 cells were infected with Ad-KLF-4 or Ad-Con virus with 1 or 5 multiplicities of infection (MOI) for 3, 6, 12, or 24 hours, and then the KLF-4 level was determined.

### 2.3. Cellular mRNA Preparation and Quantitative Analysis

Total cellular mRNA samples from LA-4 cells were prepared with the Trizol reagent (Life Technologies, Grand Island, NY, USA) under the guidance of the product's manual. Real-time quantitative reverse-transcription polymerase chain reaction (RT-qPCR) was adopted to quantify the mRNA levels of KLF-4, PAI-1, and *β*-actin with the ShinePolo One-Step RT-PCR qPCR Kit (Shanghai ShineGene Molecular Biotech, Shanghai, China) according to the kit's manual. The primers for KLF-4, PAI-1, and *β*-actin were synthesized by Sangon Biotech (Sangon, Shanghai, China). The RT-qPCR was performed on the Roche cycler 2.0 (Roche Diagnostics, Mannheim, Germany). The mRNA level for KLF-4 or PAI-1 was normalized to internal control *β*-actin and presented as the fold change over control with the ΔΔCt method [30].

### 2.4. Cellular Protein Preparation and Western Blot Analysis

Cellular protein samples were isolated with a Protein Extraction Kit (Qiagen, GmbH, Hilden, Germany) according to the kit's manual and supplemented with a protease inhibitor cocktail (Abcam, Cambridge, UK). Protein samples were separated with 10% SDS-PAGE gel and were transferred to a polyvinylidene fluoride hydrophobic membrane (Millipore, Bedford, MA, USA). The membrane was blocked with 5% skimmed milk (Solarbio, Beijing, China) overnight at 4°C to cover the nonspecific binding sites. Then the membrane was inoculated with the rabbit anti-mouse KLF-4 (BM0485, Abzoom Biolabs, Dallas, TX, USA; 1 : 300), PAI-1 (sc-8979, Santa Cruz Biotechnology, Santa Cruz, CA, USA; 1 : 200), E-cadherin (sc-7870, Santa Cruz Biotechnology, Santa Cruz, CA, USA; 1 : 500), Collagen I (ab21286, Abcam, Cambridge, UK; 1 : 200), mouse anti-mouse *α*-Smooth Muscle Actin (*α*-SMA) (sc-53142, Santa Cruz Biotechnology, Santa Cruz, CA, USA; 1 : 200), or rabbit anti-mouse *β*-actin (sc-7210, Santa Cruz Biotechnology, Santa Cruz, CA, USA; 1 : 800) at room temperature for 2 hours. The specific binding to each protein marker was presented with the incubation with the peroxidase-conjugated secondary antibody (Promega, Madison, WI, USA) and the electrochemiluminescence (ECL) detection system (Amersham, Uppsala, Sweden). The protein level was presented as a ratio to *β*-actin.

### 2.5. Statistical Analysis

Statistical analyses were performed with GraphPad Software 6 (GraphPad Software, La Jolla, CA, USA). Results were presented as mean ± SEM, and the difference between two groups was analyzed by Student's *t*-test. A *p* value less than 0.05 was considered to be statistically significant.

## 3. Results

### 3.1. TGF-*β*1 Downregulates Krüppel-Like Factor 4 in Mouse Lung Epithelial LA-4 Cells

**Figure 1:**
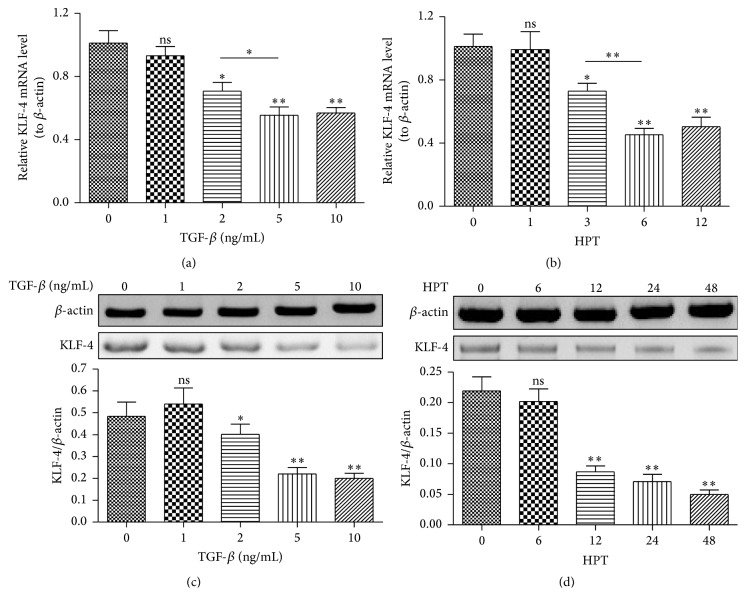
TGF-*β* downregulates KLF-4 in both mRNA and protein levels in mouse lung epithelial LA-4 cells. (a) KLF-4 mRNA level in LA-4 cells, which were treated with 0, 1, 2, 5, or 10 ng/mL TGF-*β* for 12 hours. (b) KLF-4 mRNA level in LA-4 cells after the treatment with 5 ng/mL TGF-*β* for 0, 1, 3, 6, or 12 hours. (c) Western blot analysis of KLF-4 in LA-4 cells after the treatment with 0, 1, 2, 5, or 10 ng/mL TGF-*β* for 24 hours; each KLF-4 value was expressed as percentage to *β*-actin. (d) KLF-4 in protein level in LA-4 cells after treatment with 5 ng/mL TGF-*β* for 0, 6, 12, 24, or 48 hours. Each piece of data was averaged for triple independent experiments. HPT: hours posttreatment, ^*∗*^ *p* < 0.05, ^*∗∗*^ *p* < 0.01, ^*∗∗∗*^ *p* < 0.001, or ns: no significance.

KLF-4 has been recently recognized to be downregulated during renal fibrosis and functions as a suppressor of renal fibrogenesis [14], and the antifibrosis effect of KLF-4 has also been recognized in hepatocellular carcinoma cells [15]. To investigate the possible role of KLF-4 in lung fibrosis, we firstly examined the regulation of TGF-*β* on the KLF-4 expression in mouse lung epithelial LA-4 cells. It was indicated in Figure 1(a) that the KLF-4 mRNA level was significantly downregulated in LA-4 cells which were treated with 2 to 10 ng/mL TGF-*β*, revealed by the quantitative PCR method, dose-dependently (*p* < 0.05 or *p* < 0.01). There was significant difference in KLF-4 mRNA level between the groups of 2 and 5 ng/mL or between 2 and 10 ng/mL groups (*p* < 0.05). And Figure 1(b) demonstrated that such downregulation was also time-dependent; the KLF-4 mRNA was downregulated from 3 hours after TGF-*β* treatment with 5 ng/mL and lasted to 12 hours of posttreatment (HPT) (*p* < 0.05 or *p* < 0.01), with a significant difference between the 3-hour and 6-hour treatment groups (*p* < 0.05). To reconfirm the downregulation, we also examined the KLF-4 in protein level in LA-4 cells with TGF-*β* treatment. As shown in Figures 1(c) and 1(d), Western blot analysis indicated that the TGF-*β* treatment significantly downregulated the KLF-4 protein level in LA-4 cells, dose-dependently (*p* < 0.05 or *p* < 0.01, Figure 1(c)) and time-dependently (*p* < 0.05 or *p* < 0.01, Figure 1(d)). Thus, we confirmed that TGF-*β* treatment downregulated the KLF-4 expression in mouse lung epithelial LA-4 cells.

### 3.2. TGF-*β* Promotes Plasminogen Activator Inhibitor Type-1 (PAI-1) and Induces Epithelial Mesenchymal Transition (EMT) in LA-4 Cells

**Figure 2:**
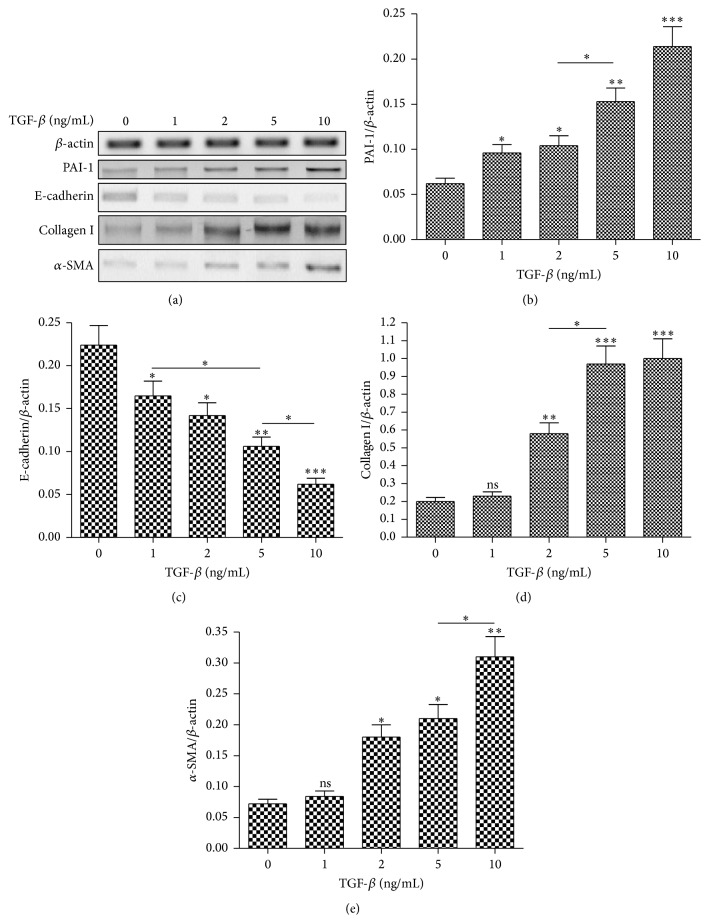
TGF-*β* promotes PAI-1 and induces epithelial-to-mesenchymal transition (EMT) in LA-4 cells. (a) Western blot analysis of PAI-1 and EMT-associated markers in LA-4 cells which were treated with 0, 1, 2, 5, or 10 ng/mL TGF-*β* for 24 hours. (b–e) Relative levels of PAI-1 (b), E-cadherin (c), Collagen I (d), and *α*-SMA (e) to *β*-actin in the LA-4 cells, which were treated with 0, 1, 2, 5, or 10 ng/mL TGF-*β* for 24 hours; each result was averaged for three independent experiments. Statistical significance was shown as ^*∗*^ *p* < 0.05, ^*∗∗*^ *p* < 0.01, ^*∗∗∗*^ *p* < 0.001, or ns: no significance.

To investigate the regulation on EMT in LA-4 cells, we examined the EMT induction by TGF-*β* in LA-4 cells. Western blotting results in Figure 2(a) indicated that the TGF-*β* treatment with 1, 2, 5, or 10 ng/mL or more markedly promoted PAI-1 (*p* < 0.05, *p* < 0.01, or *p* < 0.001, Figure 2(b)), whereas it downregulated the epithelial cell marker, E-cadherin (*p* < 0.05, *p* < 0.01, or *p* < 0.001, Figure 2(c)), in LA-4 cells, dose-dependently (*p* < 0.05). Then we examined the promotion to the other two fibrosis-associated markers, *α*-SMA and Collagen I, in the TGF-*β*-treated LA-4 cells. Both markers were also confirmed by the Western blot analysis to be promoted to significantly higher levels by the treatment with 2, 5, or 10 ng/mL TGF-*β* (*p* < 0.05, *p* < 0.01, or *p* < 0.001, Figures 2(d) and 2(e)). Taken together, TGF-*β* promotes PAI-1 and induces EMT in LA-4 cells.

### 3.3. PAI-1 Knockdown by RNAi Inhibits the TGF-*β*-Induced EMT in LA-4 Cells

**Figure 3:**
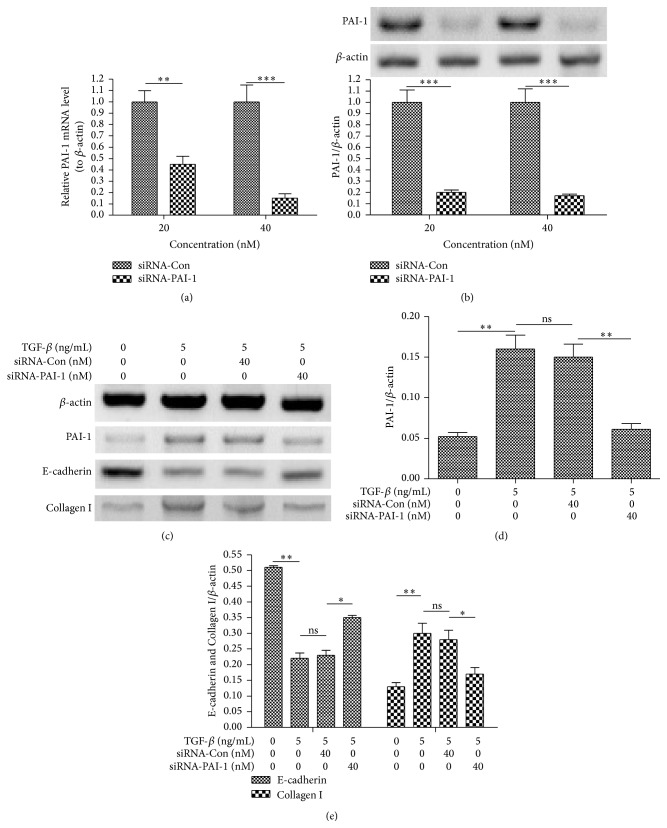
PAI-1 knockdown inhibits the TGF-*β*-promoted EMT in LA-4 cells. (a) PAI-1 mRNA level in LA-4 cells which were transfected with 20 or 40 nM PAI-1-specific siRNA (siRNA-PAI-1) or with control siRNA (siRNA-Con) for 12 hours. (b) Western blot analysis of PAI-1 in LA-4 cells which were transfected with 20 or 40 nM siRNA-PAI-1 or with siRNA-Con for 24 hours. (c) Western blot analysis of PAI-1, E-cadherin, and Collagen I in LA-4 cells which were transfected with 20 or 40 nM siRNA-PAI-1 or with siRNA-Con and were treated with 5 ng/mL TGF-*β* for 24 hours. (d) and (e) Relative levels of PAI-1 (d) or E-cadherin and Collagen I (e) in the siRNA-PAI-transfected and the TGF-*β*-treated LA-4 cells. All results were shown as values ± SE for three independent experiments. ^*∗*^ *p* < 0.05, ^*∗∗*^ *p* < 0.01, ^*∗∗∗*^ *p* < 0.001, or ns: no significance.

To confirm the role of PAI-1 in the TGF-*β*-induced EMT in LA-4 cells, the RNAi technology was utilized to abrogate the PAI-1 expression in LA-4 cells and reevaluated the EMT induction by TGF-*β* in the LA-4 cells. It was demonstrated that the PAI-1-specific siRNA (siRNA-PAI-1) transfection for 24 hours significantly reduced both mRNA (Figure 3(a)) and protein (Figure 3(b)) levels of PAI-1 (*p* < 0.01 or *p* < 0.001), compared with the control siRNA transfection. And the TGF-*β*-promoted PAI-1 was also inhibited by the siRNA-PAI-1 transfection (*p* < 0.01, Figures 3(c) and 3(d)). Moreover, the TGF-*β*-downregulated E-cadherin was markedly reversed (*p* < 0.05, Figures 3(c) and 3(d)), and the TGF-*β*-upregulated Collagen I was significantly inhibited by the siRNA-PAI-1 transfection rather than the control siRNA transfection. Therefore, the PAI-1 knockdown with siRNA-PAI-1 inhibited TGF-*β*-induced EMT in LA-4 cells.

### 3.4. KLF-4 Overexpression by Adenovirus Downregulates the TGF-*β*-Promoted PAI-1 and Inhibits the TGF-*β*-Induced EMT in LA-4 Cells

**Figure 4:**
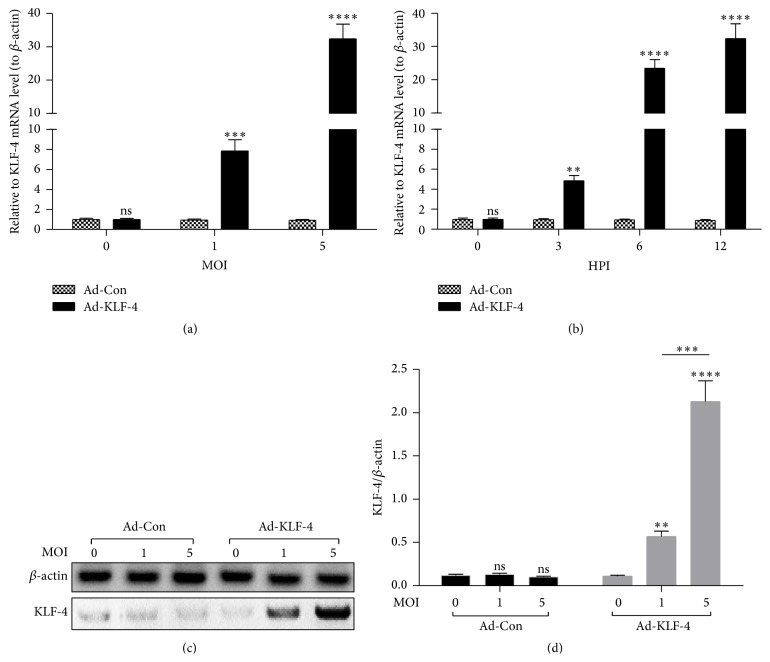
KLF-4 in both mRNA and protein levels in LA-4 cells, which were infected with KLF-4-overexpressed adenovirus. (a) The mRNA level of KLF-4 in LA-4 cells which were infected with 0, 1, or 5 multiplicity of infection (MOI) KLF-4-overexpressed (Ad-KLF-4) or control (Ad-Con) adenovirus for 12 hours. (b) KLF-4 mRNA level in LA-4 cells which were infected with 5 MOI Ad-KLF-4 or Ad-Con for 0, 3, 6, or 12 hours. (c) and (d) Protein levels of KLF-4 in LA-4 cells which were infected with 0, 1, or 5 MOI Ad-KLF-4 or Ad-Con virus. The protein level of KLF-4 was normalized to *β*-actin. Data represent the mean ± SEM for triple independent experiments. ^*∗∗*^ *p* < 0.01, ^*∗∗∗*^ *p* < 0.001, ^*∗∗∗∗*^ *p* < 0.0001, or ns: no significance.

To further identify the role of KLF-4 in the TGF-*β*-induced EMT in LA-4 cells, we then constructed a KLF-4-overexpressed adenovirus. Firstly, KLF-4 coding sequence was amplified and cloned into the shuttle plasmid. Then the recombinant adenovirus (Ad-KLF-4) was rescued via the cotransfection with the adenoviral genomic plasmid and the shuttle plasmid. The expression of KLF-4 was evaluated in LA-4 cells which were infected with Ad-KLF-4.  Figure 4(a) indicated that the Ad-KLF-4 infection with a multiplicity of infection (MOI) of 1 or 5 dramatically promoted the KLF-4 mRNA level, at 12-hour postinfection (HPI) (*p* < 0.001 or *p* < 0.0001). And the promotion was significant since 3-hour postinfection and peaked at 12-hour postinfection (*p* < 0.0001, Figure 4(b)). And the KLF-4 promotion was also confirmed in protein level (*p* < 0.01, *p* < 0.001, or *p* < 0.0001; Figures 4(c) and 4(d)).

**Figure 5:**
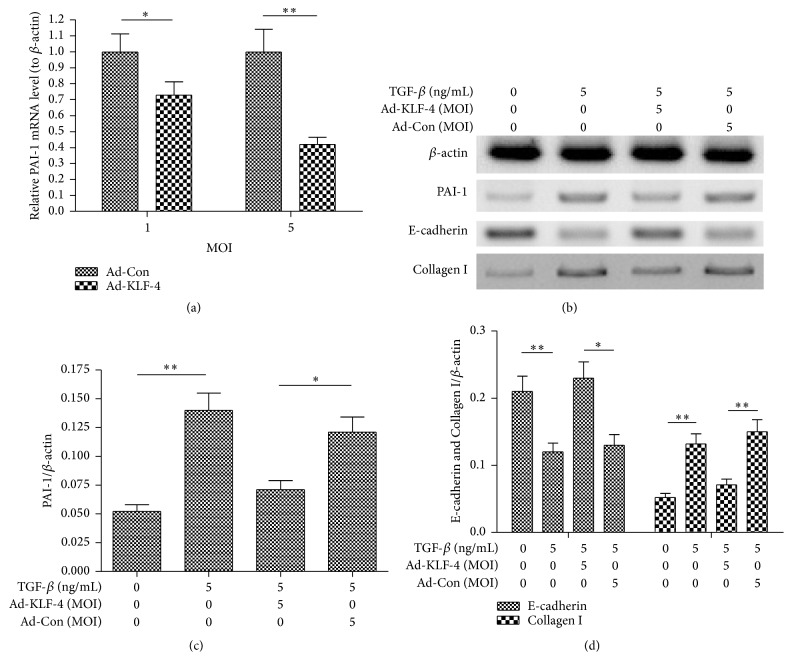
Adenovirus-mediated KLF-4 overexpression inhibits the TGF-*β*-promoted EMT in LA-4 cells. (a) PAI-1 mRNA level in the LA-4 cells which were infected with 1 or 5 multiplicity of infection (MOI) KLF-4-overexpressed adenovirus (Ad-KLF-4) or control adenovirus (Ad-Con) for 12 hours. (b) Western blot analysis of PAI-1, E-cadherin, and Collagen I in LA-4 cells which were treated with 5 ng/mL TGF-*β* and were infected with 1 or 5 MOI Ad-KLF-4 or Ad-Con for 24 hours. (c) and (d) Relative levels of PAI-1 (c) or E-cadherin and Collagen I (d) in the Ad-KLF-4- or Ad-Con-infected and the TGF-*β*-treated LA-4 cells (5 MOI). Each value was averaged for triple independent results. ^*∗*^ *p* < 0.05, ^*∗∗*^ *p* < 0.01.

To investigate the regulation of KLF-4 overexpression on the TGF-*β*-induced PAI-1 and EMT in LA-4 cells, we reevaluated the expression of PAI-1 in both mRNA and protein levels and the levels of E-cadherin and Collagen I in the TGF-*β*-treated LA-4 cells. It was shown in Figure 5(a) that the PAI-1 mRNA level was significantly reduced in the Ad-KLF-1-infected LA-4 cells, than in the Ad-Con-infected LA-4 cells (*p* < 0.05 or *p* < 0.01 for 1 or 5 MOI). And the Western blotting results demonstrated that the PAI-1 in protein level was also markedly downregulated by the Ad-KLF-1 infection with 5 MOI (*p* < 0.05, Figures 5(b) and 5(c)). Moreover, the Ad-KLF-1 infection with 5 MOI also markedly ameliorated the E-cadherin inhibition by the TGF-*β* treatment (*p* < 0.05, Figure 5(d)), and the TGF-*β*-upregulated Collagen I was significantly inhibited by the Ad-KLF-1 infection with 5 MOI. Therefore, KLF-4 overexpression by adenovirus downregulates the TGF-*β*-promoted PAI-1 and inhibits the TGF-*β*-induced EMT in LA-4 cells.

## 4. Discussion

EMT is increasingly recognized as an important pathway in the lung fibrosis, via giving rise to fibroblasts and myofibroblasts. The most convincing evidence for EMT as a source of myofibroblasts* in vivo* is the murine models of pulmonary fibrosis [31]. The contribution of EMT to fibroblasts is estimated up to one-third of total fibroblasts [32, 33]. The transition from epithelial cells into myofibroblasts is controlled via several complex mechanisms, in which TGF-*β* is one of the most potent EMT mediators [34, 35]. However, the orchestrated mechanism underlining the TGF-*β*-induced EMT in lung fibrosis is far from clearness.

In this study, we constructed the model of TGF-*β*-induced EMT in mouse LA-4 lung epithelial cells. E-cadherin was markedly reduced, whereas Collagen I and *α*-SMA were significantly promoted by the TGF-*β* treatment. And PAI-1 was upregulated, whereas KLF-4 was downregulated in the TGF-*β*-induced EMT model in LA-4 cells. We also confirmed the key mediatory role of PAI-1 in the TGF-*β*-induced EMT in such model; the siRNA-mediated PAI-1 knockdown inhibited the TGF-*β*-induced EMT, whereas the adenovirus-medicated KLF-4 overexpression markedly reduced the TGF-*β*-induced PAI-1 and significantly inhibited the TGF-*β*-induced EMT in LA-4 cells. Our study for the first time confirmed that the downregulation of KLF-4 contributed the TGF-*β*-induced EMT in LA-4 cells. And it was indicated that the KLF-4 overexpression inhibited the TGF-*β*-induced EMT via downregulating PAI-1 in LA-4 cells.

Recent studies have identified a novel and specific DNA sequence in the PAI-1 promoter, referred to as nonconsensus xenobiotic response element (NC-XRE), to which aryl hydrocarbon receptor (AhR) binds [36]. And it is demonstrated that the AhR and KLF-6 proteins form an obligatory heterodimer necessary for NC-XRE binding; NC-XRE is a recognition site for AhR and KLF-6, via* in vivo* chromatin immunoprecipitations and* in vitro* DNA binding studies [37]. And KLF-2 and KLF-4 are also well recognized to target the promoter of PAI-1 in endothelial cells [38, 39]. The present study indicated that both KLF-4 and PAI-1 were implicated in the TGF-*β*-induced EMT in LA-4 cells. And the negative regulation by KLF-4 on the expression of PAI-1 was also confirmed in the LA-4 lung epithelial cells, implying the targeting inhibition of PAI-1 by KLF-4 in such type of cells. However, it needs to be investigated in more detail about the PAI-1 expression by KLF-4. And other possible targets, which might be involved in the EMT or lung fibrosis, need to be investigated.

In conclusion, our study confirmed the downregulation of KLF-4 in the TGF-*β*-induced EMT in LA-4 cells. And the KLF-4 overexpression significantly reduced the TGF-*β*-induced PAI-1 and thus inhibited the TGF-*β*-induced EMT in mouse lung epithelial LA-4 cells. The present study implied that KLF-4 might be a promising target for effective control of the pulmonary fibrosis.
